# Inhibitory effect of caveolin-1 in vascular endothelial cells, pericytes and smooth muscle cells

**DOI:** 10.18632/oncotarget.19191

**Published:** 2017-07-12

**Authors:** Hongping Xu, Liwei Zhang, Wei Chen, Jiazhou Xu, Ruting Zhang, Ran Liu, Lan Zhou, Wenjie Hu, Rong Ju, Chunsik Lee, Weisi Lu, Anil Kumar, Xuri Li, Zhongshu Tang

**Affiliations:** ^1^ State Key Laboratory of Ophthalmology, Zhongshan Ophthalmic Center, Sun Yat-sen University, Guangzhou 510060, P. R. China

**Keywords:** caveolin-1, cavtratin, endothelial cell, pericyte, smooth muscle cell

## Abstract

Caveolin-1 (Cav1) is the principle structural protein of caveolae. It plays important roles in the vascular system under both physiological and pathological conditions. Although Cav1 has been shown to inhibit microvascular permeability and has been considered as a tumor-suppressor for years, the underlying cellular mechanism has yet to be discovered. Here, we systematically investigated Cav1 functions in the main types of vascular cells, including endothelial cells (ECs), pericytes (PCs) and smooth muscle cells (SMCs). We synthesized a cell-permeable peptide called cavtratin that is derived from the Cav1 scaffolding domain. We found that cavtratin inhibited ECs in all assays, including survival, proliferation, migration and permeability assays. It also inhibited the proliferation of PCs and SMCs but had no effect on their survival or migration. The inhibitory effect of cavtratin on the proliferation of all vascular cells suggests that Cav1 plays important roles in vascular development and angiogenesis. Under physiological condition, the main function of Cav1 is to inhibit EC permeability.

## INTRODUCTION

Caveola is a flask-shaped vesicular structure located near the plasma membrane. It is involved in many important cellular processes, such as transcellular transport and signal transduction [1, 2]. Cav1 is the main structural protein of caveolae. In the vasculature, Cav1 is abundant in endothelial cells (ECs) and less abundant in smooth muscle cells (SMCs) and pericytes (PCs) [3, 4]. The main functional domain of Cav1 is a 20-aa scaffolding domain (CSD), which mediates interactions between Cav1 and other proteins such as TrkA, EGFR, Neu/ErbB2, H-Ras, MEK, c-Src, Fyn, GPCRs, and eNOS [5–8]. A 36-aa peptide called cavtratin is a fused peptide of the scaffolding domain with a short 16-aa internalization sequence (AP) from the Drosophila transcription factor antennapedia. Cavtratin has been show to enter and punctate in the vascular endothelial cells [9]. Currently, most studies indicate that cavtratin works as an analog of Cav1 to rescue or correct Cav1 deficient phenotypes, although theoretically it can also be a dominant negative of Cav1 [10–12].

Cavtratin has been shown to reduce tumor vasculature and microvascular hyperpermeability and to attenuate tumor growth. Thus, cavtratin is considered a promising candidate for the inhibition of tumor progression, and *Cav1* is considered a putative tumor-suppressor gene [1, 8, 11, 13–15]. Mechanistically, inhibition of VEGFR-2 and inhibition of eNOS-dependent vascular leakage may all be related to the tumor-suppressive function of Cav1 [11, 13]. In addition, Cav1 acts on various other signaling molecules, such as epidermal growth factor, Src family tyrosine kinases and the insulin receptor [16–18]. Despite the studies on Cav1 function and molecular mechanism, little has been reported about its function in vascular cells, especially at the cellular level. However, a comprehensive study at the cellular level is important for the understanding of Cav1 function and it is also important to explore the cell-targets of Cav1-derived drugs such as cavtratin.

The major cellular components of the vasculature include the vascular ECs, the PCs and the vascular SMCs. The entire blood vessel lumen is enclosed by ECs. PCs cover ECs at the capillary level, where most of the nutrient and oxygen exchange happens. SMCs cover arteries and veins. Survival, proliferation and migration of all of the vascular cells are crucial during development and pathological conditions [19, 20]. As ECs, but not PCs or SMCs, enclose the entire blood vessel lumen and control the transport of substances in and out of the vascular system, permeability is generally considered as an aspect of ECs only.

To systematically explore the effect of Cav1 on the vasculature, we applied cavtratin to the main vascular cells and evaluated the survival, proliferation, and migration of all of these cells and the permeability of ECs. We found that cavtratin inhibited ECs in all aspects including survival, proliferation, migration and permeability. In addition, it also inhibited the proliferation of PCs and SMCs but did not have any effect on their survival or migration.

## RESULTS

### Cavtratin inhibits the survival of endothelial cells

**Figure 1:**
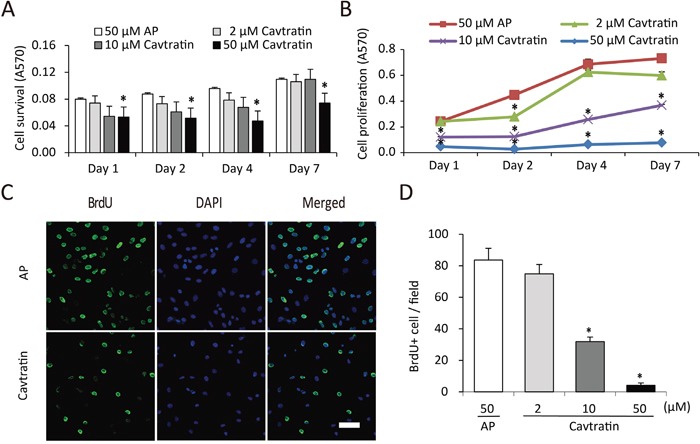
Cavtratin inhibits the survival and proliferation of endothelial cells **(A)** Cavtratin decreased HUVEC survival in an MTT assay with 0.5% of FBS in the culture medium. Cells were treated with different concentrations (2 μM, 10 μM and 50 μM) of cavtratin or with 50 μM AP as a control. Absorbance at 570 nm (A570) indicated the cell survival. **(B)** HUVEC proliferation was arrested under treatment with cavtratin in an MTT assay with 10% of FBS in the culture medium. Cells were treated with 2 μM, 10 μM or 50 μM cavtratin, or with 50 μM AP as a control. A570 represented the cell proliferation. **(C, D)** Treatment with cavtratin significantly decreased BrdU incorporation into HUVECs. Cells were treated with 50 μM cavtratin or AP for 4 days. BrdU was also added to the medium to label the proliferating cells, which were later visualized by anti-BrdU staining. Images were captured under the same confocal setting. All BrdU+ cells were counted, including those with weak fluorescence. All data represents the mean ± SEM. *, P < 0.05 versus the AP group under the same conditions. Scale bar, 50 μm.

Vascular endothelial cells form the inner surface of blood vessels, and thus, they are important for vascular function. To determine the effect of cavtratin on endothelial cells, firstly we examined the survival of human umbilical vein endothelial cells (HUVECs) under a minimal level of serum. The cells were cultured in endothelial cell medium supplemented with 0.5% FBS and 2 μM, 10 μM or 50 μM cavtratin or 50 μM AP as a control. A MTT assay was performed to evaluate cell survival. The results indicated that the treatment with cavtratin led to a 49% to 67% decrease of the survival of ECs (Figure 1A). The MTT assay in 0.5% FBS revealed that cavtratin inhibits the survival of endothelial cells.

### Cavtratin inhibits the proliferation of endothelial cells

Then we increased the concentration of serum in the medium to 10% and did the MTT assay again. As shown in Figure 1B, the cell population kept increasing in all groups from day 1 to day 7, but the cell numbers in the groups treated with cavtratin of different concentrations were only approximately 26% to 69% of the cell numbers in the AP group. This result suggested that cavtratin inhibits HUVEC proliferation too. To confirm that effect, we performed a BrdU incorporation assay. BrdU was added to the medium to label proliferating cells at the same time as cavtratin or AP was added and was incubated for 4 days. Anti-BrdU immunostaining demonstrated that cavtratin treatment led a significant decrease in BrdU incorporation (Figure 1C). The number of BrdU^+^ cells in the 50 μM cavtratin group was approximately only 9% of that in the AP group (Figure 1D). Altogether, both the MTT and BrdU incorporation assays in 10% FBS indicate that cavtratin inhibits the proliferation of endothelial cells.

### Cavtratin inhibits the migration of endothelial cells

**Figure 2:**
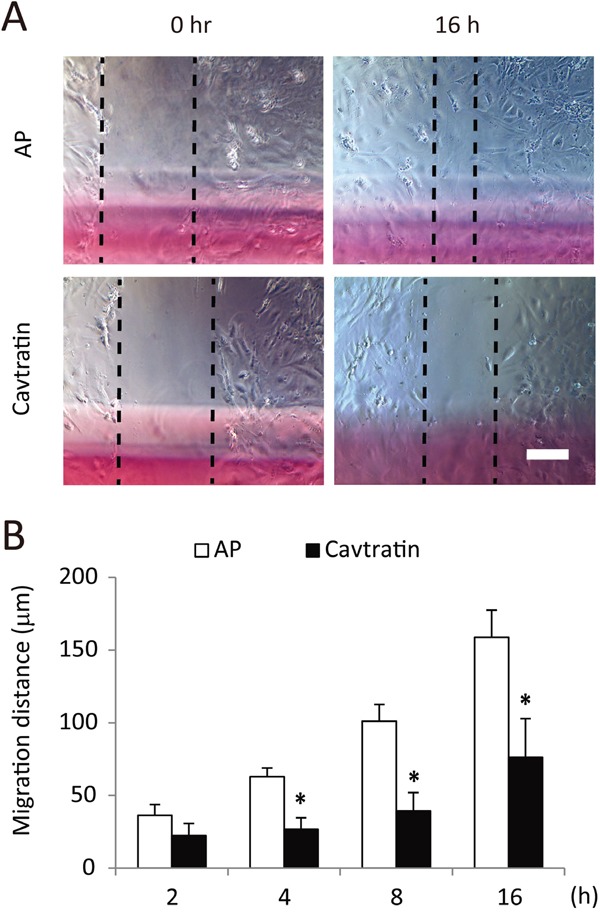
Cavtratin inhibits the migration of endothelial cells **(A)** Wound healing assay with HUVECs. A linear wound was made across the cell monolayer, and 50 μM cavtratin or AP was added to the culture medium. The distance between the cell boundaries was measured at different time points. **(B)** Statistical analysis of HUVEC migration. *, P < 0.05 versus the AP group under the same conditions. Scale bar, 200 μm.

Next, we assessed the effect of cavtratin on endothelial cell migration in a monolayer wound healing assay. A wound was made by scratching the cell monolayer. HUVEC migration following the addition of cavtratin or AP was analyzed at different time points. As shown in Figure 2A and 2B, HUVECs in the cavtratin (50 μM) group migrated at approximately 60% of the rate at which the cells did in the AP group. The results demonstrate that cavtratin attenuates the migration of endothelial cells.

### Cavtratin inhibits the permeability of endothelial cells

**Figure 3:**
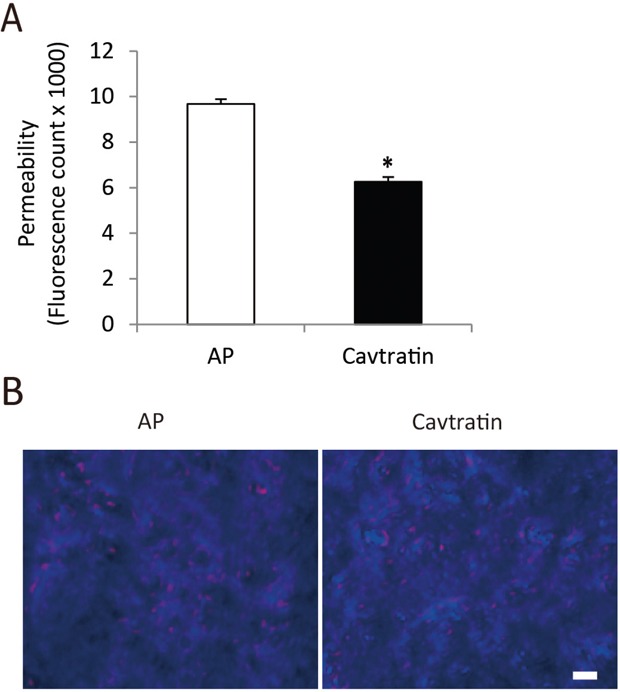
Cavtratin inhibits the permeability of endothelial cells Endothelial cell culture medium containing 50 μM cavtratin or AP was added to the mouse pulmonary microvascular endothelial cells for the *in vitro* vascular permeability assay. **(A)** Measurement of the fluorescence molecules that permeated through the cell monolayer. **(B)** Staining of cell layers showing the confluent cell monolayer. *, P<0.05 versus the AP group. Scale bar, 20 μm.

We subsequently examined the effect of cavtratin on the permeability of endothelial cells. Permeability is an important feature of ECs. To obtain cells that are very close to the physiological condition, we cultured primary ECs from mouse pulmonary microvasculature and performed the cell permeability assay *in vitro* by quantifying FITC-dextran leakage. As shown in Figure 3A, cell permeability in the cavtratin-treated group decreased to approximately 35% of that in the AP-treated group. Crystal violet staining of the cells after the permeability assay confirmed that the cells indeed formed a confluent monolayer (Figure 3B). The results thus show that cavtratin suppresses the permeability of endothelial cells.

### Cavtratin inhibits the proliferation but not the survival or migration of pericytes

**Figure 4:**
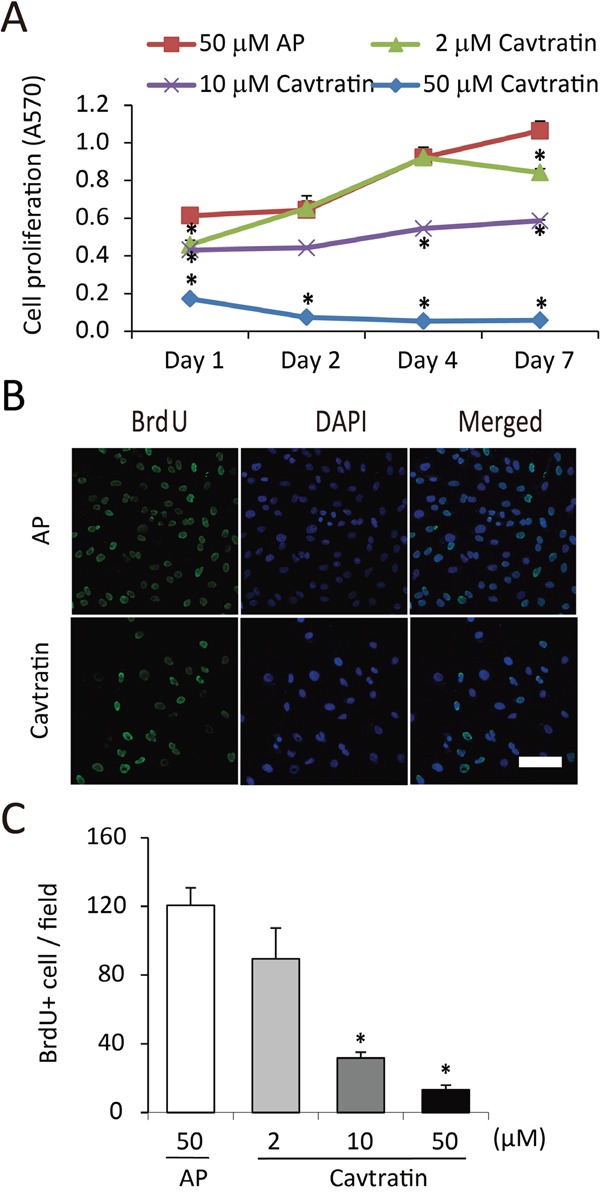
Cavtratin inhibits the proliferation of pericytes **(A)** MTT assay of human brain vascular pericytes (HBVPs) cultured in medium containing 10% FBS. The cells were treated with different concentrations of cavtratin or AP for different times as indicated. A570 indicated the cell proliferation. **(B** and **C)** BrdU incorporation assay in HBVPs. Cells were treated with 10 μM BrdU and 50 μM AP or cavtratin for 4 days. BrdU immunostaining was performed to show the proliferating cells. *, P<0.05 versus the AP group under the same conditions. Scale bar, 50 μm.

Pericytes wrap around the endothelial cells of capillaries and venules. They are also important in the development and maintenance of blood vessels [21, 22]. Pericyte proliferation, migration and survival are important processes in angiogenesis. As they do not cover the entire lumen, permeability is not a function associated with pericytes. For the proliferation assay, we cultured human brain vascular pericytes (HBVPs) in medium containing 10% FBS to keep the cells in proliferative state and examined their proliferation using an MTT assay. We found that cavtratin treatment significantly decreased pericyte proliferation compared with AP treatment (Figure 4A). We further verified the inhibitory effect in a BrdU incorporation assay and found that cavtratin markedly prohibited BrdU incorporation (Figure 4B and 4C).

For the survival assay, we cultured HBVPs in 0.5% FBS to arrest cell proliferation and evaluated cell survival using the MTT assay. Differences in pericyte survival were not observed between the AP group and groups treated with 2 to 50 μM cavtratin at different time points from day 1 to day 7 (Supplementary Figure 1A). We also performed a monolayer cell wound healing assay to examine whether cavtratin affects pericyte migration. No effect was observed regarding pericyte migration either (Supplementary Figure 1B, 1C). Altogether, the results from the HBVP experiments demonstrate that cavtratin inhibits pericyte proliferation, but it has no effect on their survival or migration.

### Cavtratin inhibits the proliferation but not the survival or migration of vascular smooth muscle cells

**Figure 5:**
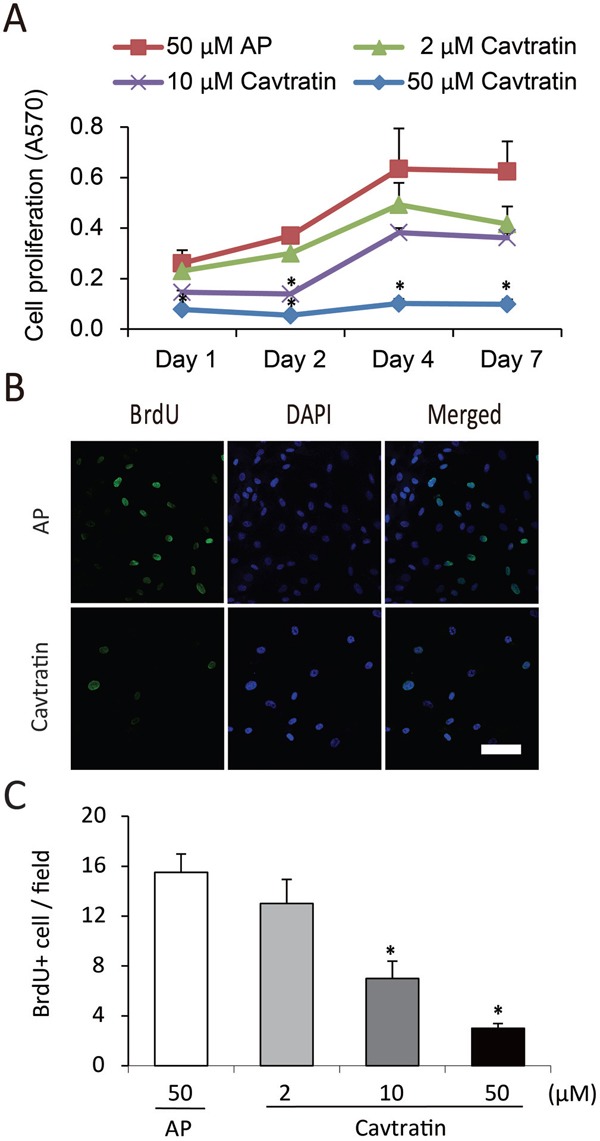
Cavtratin inhibits the proliferation of vascular smooth muscle cells **(A)** MTT assay using human umbilical vein smooth muscle cells (HUVSMCs) cultured with 10% FBS. The cells were treated with cavtratin or AP at different concentrations for different times as indicated. A570 represented the cell proliferation. **(B** and **C)** Anti-BrdU staining of HUVSMCs 4 days after BrdU and cavtratin or AP application. *, P<0.05 versus the AP group under the same conditions. Scale bar, 50 μm.

Vascular smooth muscle cells are also important components of blood vessels and have critical functions in regulating blood vessel diameters [23]. Similar to pericytes, permeability is not associated with SMCs. We therefore investigated the effect of cavtratin on SMC proliferation, migration and survival. Briefly, in an MTT assay using cells cultured in medium containing 10% FBS, we found that cavtratin treatment markedly decreased the proliferation of human umbilical vein smooth muscle cells (HUSMCs) (Figure 5A), which was further verified in a BrdU incorporation assay (Figure 5B, 5C). However, in either the survival or the migration assays, cavtratin treatment had no detectable effect on SMCs (Supplementary Figure 2). Thus, cavtratin inhibits the proliferation of smooth muscle cells but does not have any effect on their survival or migration.

## DISCUSSION

Although Cav1 has been considered as a putative tumor-suppressor and cavtratin is proposed as a drug candidate for delaying tumor progression, the target of cavtratin and their functions in different types of vascular cells are not well understood. In this study, we applied cavtratin to the main types of vascular cells and evaluated its effects on cell survival, proliferation, migration and permeability. Our results demonstrate two characteristics of Cav1 function in the vasculature: 1. Cav1 suppresses the proliferation of all vascular cells; and 2. Cav1 mainly functions in ECs. Cavtratin suppressed ECs in all assays, but it inhibited only the proliferation of PCs and SMCs without any effect on their survival or migration. Comparing with the previous research which mainly focused on the role of Cav1 in vascular permeability, our study provided a systematic understanding of the cellular basis of the anti-angiogenesis function of Cav1 and found the cellular targets of cavtratin in the treatment of neovascular diseases.

The gross inhibitory effect of cavtratin on the proliferation of all vascular cells suggests that Cav1 has a comprehensive role in vascular development and angiogenesis. Here, angiogenesis refers to the abnormal growth of new blood vessels growing from preexisting ones. It is essential in the development of vascular diseases such as tumors and diabetic retinopathy [20, 24, 25]. The angiogenesis process involves the following events: vasodilation, increased vascular permeability, extracellular matrix degradation, vascular cell proliferation, migration, invasion, and peri-endothelial maturation [26, 27]. Of these events, vascular cell proliferation is crucial to achieve sufficient cells for the formation of new vessels. Our study revealed that cavtratin inhibits the proliferation of all three types of vascular cells, thus providing the cellular basis for its anti-angiogenesis effect.

Our findings also show that cavtratin affects ECs and PCs/SMCs differently. Different cellular effects come from different mechanisms. In general, Cav1 has diverse functions that include, but not limit to, endocytosis, lipid regulation, compartmentalization of signaling pathways and calcium signaling, regulation of lipids, organization of plasma membrane domains and junctions, control of protein subcellular localization, intracellular compartmentalization, plasma membrane organization and signaling, and remodeling of the ECM [28]. As our study focused on the function of Cav1 in the main types of vascular cells at the cellular level, it did not include any molecular mechanism. The underlying molecular mechanism can be partly speculated from the publications. It is well-known that Cav1 negatively regulates endothelial nitric oxide synthase (eNOS), and thus inhibits endothelial permeability [28]. The Cav1-eNOS pathway is therefore very likely to be the mechanism of cavtratin prohibition of HUVEC permeability. At present, we are not sure about the exact molecular mechanisms of the other effects. At the molecular level, besides the well known eNOS, Cav1 has been found to interact with many receptors and intracellular molecules, which means it takes parts in many signaling pathways. To elucidate the molecular mechanism, we need to identify which molecules are involved in the process and to find their interaction partners in special pathways that lead to the special function. A future direction would be to identify such molecules, to find their interacting partners and to confirm the signal pathways even including some new pathways in which they are involved.

We did not detect any effect on the survival or migration of PCs or SMCs. DeWever *et al.* have found that Cav1 impairs the migration of smooth muscle cells and pericytes [29]. They used a multipotent stem cell line 10T1/2 in their experiments [30], but we used the mature cells HUVSMCs and HBVPs. The different results suggest that Cav1 may affect immature and mature PCs and SMCs differently.

This study suggests that all of the vascular cells are the target of cavtratin after it is applied *in vivo*, with an overall inhibition but different effects on different types of vascular cells. In this study, all the assays were conducted *in vitro*. It is still an open question whether these results faithfully resemble the situation of angiogenesis *in vivo*. Further study needs to be conducted in animals to verify the cell culture results, which suggests another direction for the future work.

## MATERIALS AND METHODS

### Main reagents

Cavtratin (RQIKIWFQNRRMKWKKDGIWKASFTTFTVTKYWFYR) and AP (RQIKIWFQNRRMKWKK) were synthesized by GL Biochem, Shanghai, China. The powder was dissolved in 1 mM acetic acid, aliquoted and diluted to 10 mM with distilled water before experiments.

### Cell culture

The primary mouse endothelial cells were prepared from mouse lungs and purified with mouse CD31 MicroBeads (Miltenyi Biotec) according to the product manual with modifications. Briefly, mice were transcardially perfused with saline, and the lungs were isolated, diced into fine pieces and transferred to a flask coated with poly-ornithine (Sigma) with a small amount of FBS. The flask was kept upside down in an incubator for 2 hours, then returned to the normal position and filled with endothelial cell medium supplemented with 100 U/ml penicillin–streptomycin and 20% FBS. After the cells spreaded from the pellets and grew up to 60-70% confluence, the pellets were transferred to a new flask. The cell layer was trypsinized and resuspended in fresh medium. The antibody-coated dynabeads were added, and the endothelial cells bound to the beads were collected with a magnet and cultured for further experiments.

Human umbilical vein endothelial cells (HUVECs), human brain vascular pericytes (HBVPs), and human umbilical vein smooth muscle cells (HUVSMCs) were purchased from ScienceCell (Shanghai, China).

### MTT assay

The MTT assay was conducted for cell survival and proliferation studies according to the product manual with modifications (Molecular Probe). Cells were cultured in medium containing 0.5% and 10% of FBS for the survival and proliferation studies, respectively. Cavtratin or AP was added, and 1, 2, 4 or 7 days later, MTT (3-(4,5-dimethylthiazol-2-yl)-2,5-diphenyltetrazolium bromide) was added to the medium and incubated for 4 hours. The formazan product was solubilized in 2-isopropanol with 0.1 M HCl and measured at 570 nm.

### BrdU incorporation assay for cell proliferation

Cells were passaged into a 96-well plate. Twenty-four hours later, BrdU (Sigma, B5002) was added at a working concentration of 10 μM to label the proliferating cells. For immunostaining, cells were fixed in 4% paraformaldehyde for 10 minutes followed by incubation with 2 M HCl at 37 °C for 30 minutes before neutralizing with 0.1 M borate buffer (pH=8.5). After blocking and permeabilizing in 0.01 M PBS with 5% serum and 0.5% Triton-X-100 for 30 minutes, a sheep anti-BrdU primary antibody (1:500, Novus, NB500-235) and a fluorescence-conjugated secondary antibody were applied sequentially, with 3 washes after each antibody incubation step. Cells were observed under a Zeiss microscope. BrdU-positive cells were counted. Eight fields were collected from each group for statistical analysis.

### Wound healing assay for cell migration

Cells were plated in a 48-well plate and grown until a confluent layer was formed. A linear wound of approximately 0.5-mm wide was made across the well. Cells were washed twice to remove the detached cells and debris. The distance between both cell boundaries was measured at different time points.

### *In vitro* vascular permeability assay

A Millipore *in vitro* vascular permeability assay kit (Millipore, ECM644) was used for the permeability assay following the product instructions. A high molecular weight FITC-Dextran, as described in the manual book, was supposed to pass through a HUVEC monolayer according to the permeability of the cells. Briefly, cells were plated in a permeability insert and incubated until a monolayer was formed. Then, the insert was transferred to a fresh well and the medium was exchanged with fresh medium supplemented with 50 μM AP or cavtratin. Ten hours later, the insert was transferred again to another fresh well, and medium containing FITC-Dextran was added to each insert and incubated for 20 minutes. A 100-μl volume of the medium in the receiver tray was removed and read under 485 nm and 535 nm as the excitation and emission wavelengths, respectively. The cell monolayer was further stained with crystal violet and observed to determine the cell monolayer integrity.

### Statistical analysis

For each assay, the cells were passaged and cultured under the same condition. Statistics was carried out in quadruplicate for each group. The results are expressed as mean ± SEM (Standard Error of the Mean). The differences between the experimental groups and the control groups were analyzed using t-tests with significance set at *, P<0.05, using Microsoft Excel. Each experiment was repeated at least three times independently.

## SUPPLEMENTARY MATERIALS AND FIGURES



## Abbreviations

AP: antennapedia
ECs: endothelial cells
HBVPs: human brain vascular pericytes
HUVECs: human umbilical vein endothelial cells
HUVSMCs: human umbilical vein smooth muscle cells
PCs: pericytes
SMCs: smooth muscle cells
MTT: 3-(4,5-dimethylthiazol-2-yl)-2,5- diphenyltetrazolium bromide
