# Supplementary material for: Inhibitory effect of caveolin-1 in vascular endothelial cells, pericytes and smooth muscle cells
Source: Oncotarget. 2017 Jul 12;8(44):76165–73. doi: 10.18632/oncotarget.19191 (PMC5652695; doi:10.18632/oncotarget.19191)
Supplement: Supplementary file 1 [file oncotarget-08-76165-s001.pdf]

Inhibitory effect of caveolin-1 in vascular endothelial cells, pericytes and smooth muscle cells

SUPPLEMENTARY MATERIALS

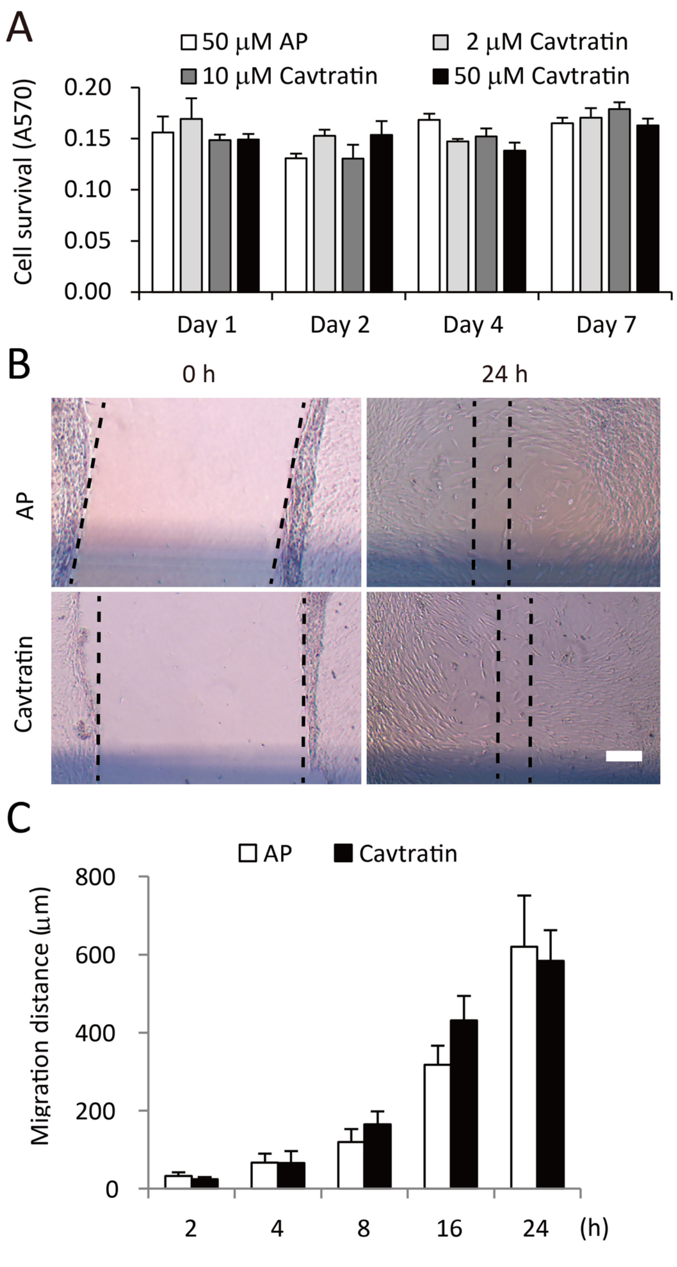

Supplementary Figure 1: Cavtratin has no effect on the survival or migration of pericytes. (A) MTT assay of HBVPs cultured with 0.5% serum in the medium. Cells were treated with different concentrations of cavtratin or AP as indicated. A570 indicated the cell survival level. **(B** and **C)** Wound healing assay for HBVP migration. Either 50 μM cavtratin or AP was applied. Scale bar, 100 μm.

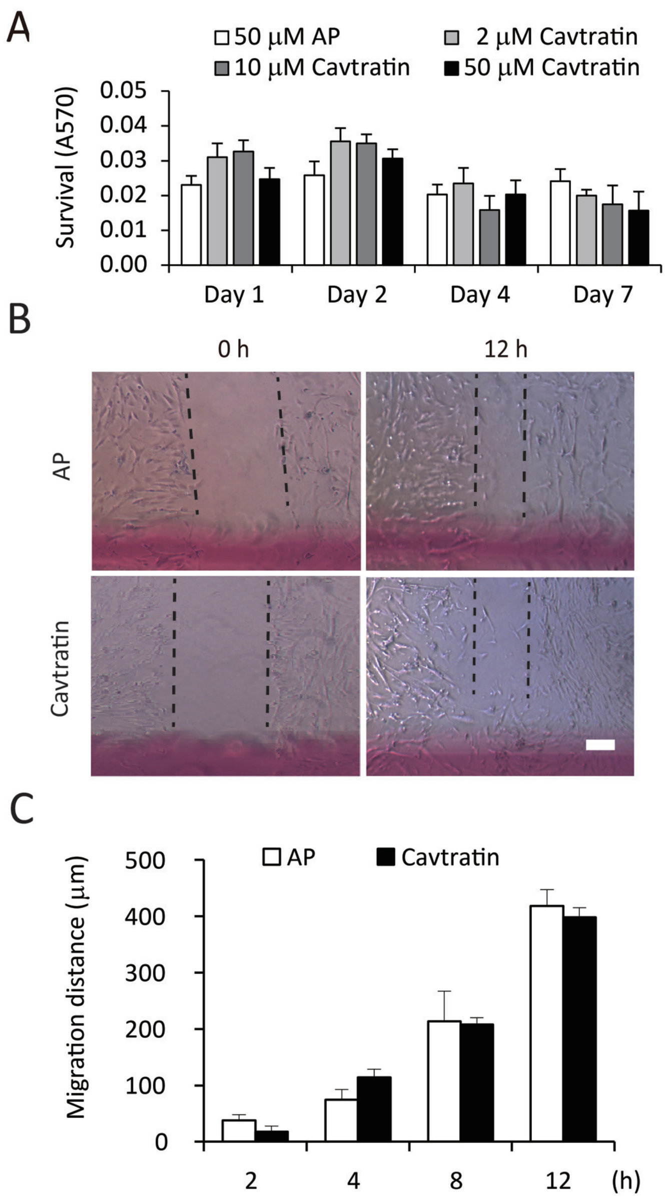

Supplementary Figure 2: Cavtratin has no effect on the survival or migration of smooth muscle cells. **(A)** MTT assay using HUVSMCs cultured with 0.5% serum. Cells were treated with cavtratin or AP. A570 was used as an indicator of cell survival. **(B** and **C)** Wound healing assay for HUVSMC migration. Scale bar, 200 μm.
